# Luna Virus and Helminths in Wild *Mastomys natalensis* in Two Contrasting Habitats in Zambia: Risk Factors and Evidence of Virus Dissemination in Semen

**DOI:** 10.3390/pathogens11111345

**Published:** 2022-11-14

**Authors:** Samuel Munalula Munjita, Given Moonga, Andrew Nalishuwa Mukubesa, Joseph Ndebe, Benjamin Mubemba, Manu Vanaerschot, Cristina Tato, John Tembo, Nathan Kapata, Simbarashe Chitanga, Katendi Changula, Mashiro Kajihara, Walter Muleya, Ayato Takada, Elisabeth Fichet-Calvet, Alimuddin Zumla, Hirofumi Sawa, Matthew Bates, Sody Munsaka, Edgar Simulundu

**Affiliations:** 1 Department of Biomedical Sciences, School of Health Sciences, University of Zambia, Lusaka 10101, Zambia; 2 Department of Disease Control, School of Veterinary Medicine, University of Zambia, Lusaka 10101, Zambia; 3 Africa Center of Excellence for Infectious Diseases of Humans and Animals, University of Zambia, Lusaka 10101, Zambia; 4 Department of Epidemiology and Biostatistics, School of Public Health, University of Zambia, Lusaka 10101, Zambia; 5 Department of Wildlife Sciences, School of Natural Resources, Copperbelt University, Kitwe 50100, Zambia; 6 Department of Biomedical Sciences, School of Medicine, Copperbelt University, Ndola 50100, Zambia; 7 Chan-Zuckerberg Biohub, San Francisco, CA 94158, USA; 8 HerpeZ, University Teaching Hospital, Lusaka 10101, Zambia; 9 Zambia National Public Health Institute, Ministry of Health, Lusaka 10101, Zambia; 10 Department of Paraclinical Studies, School of Veterinary Medicine, University of Namibia, Windhoek 10005, Namibia; 11 School of Life Sciences, College of Agriculture, Engineering and Sciences, University of KwaZulu-Natal, Durban 4000, South Africa; 12 Department of Paraclinical Studies, School of Veterinary Medicine, University of Zambia, Lusaka 10101, Zambia; 13 Division of Global Epidemiology, International Institute for Zoonosis Control, Hokaido University, Sapporo 001-0020, Japan; 14 Department of Biomedical Sciences, School of Veterinary Medicine, University of Zambia, Lusaka 10101, Zambia; 15 Department of Virology/Disease Ecology, Bernhard-Nocht Institute for Tropical Medicine, Bernhard-Nocht Strasse 74, 20359 Hamburg, Germany; 16 Division of Infection and Immunity, Centre for Clinical Microbiology, University College London, NIHR Biomedical Research Centre, University College London Hospitals NHS Foundation Trust, London NW3 2PF, UK; 17 International Institute for Zoonosis Control, Hokkaido University, Sapporo 001-0020, Japan; 18 International Collaboration Unit, Global Virus Network, Baltimore, MD 21201, USA; 19 One Health Research Center, Hokkaido University, Sapporo 001-0020, Japan; 20 Institute for Vaccine Research and Development (HU-IVReD), Hokkaido University, Sapporo 001-0020, Japan; 21 Joseph Banks Laboratories, School of Life and Environmental Sciences, University of Lincoln, Lincolnshire LN6 7TS, UK; 22 Macha Research Trust, Choma 20100, Zambia

**Keywords:** metagenomics, semen, foetus, reads, prevalence, risk factors, Luna virus, rodents, *Mastomys natalensis*, Zambia

## Abstract

Transmission dynamics and the maintenance of mammarenaviruses in nature are poorly understood. Using metagenomic next-generation sequencing (mNGS) and RT-PCR, we investigated the presence of mammarenaviruses and co-infecting helminths in various tissues of 182 *Mastomys natalensis* rodents and 68 other small mammals in riverine and non-riverine habitats in Zambia. The Luna virus (LUAV) genome was the only mammarenavirus detected (7.7%; 14/182) from *M. natalensis*. Only one rodent from the non-riverine habitat was positive, while all six foetuses from one pregnant rodent carried LUAV. LUAV-specific mNGS reads were 24-fold higher in semen than in other tissues from males. Phylogenetically, the viruses were closely related to each other within the LUAV clade. Helminth infections were found in 11.5% (21/182) of *M. natalensis*. LUAV–helminth co-infections were observed in 50% (7/14) of virus-positive rodents. Juvenility (OR = 9.4; *p* = 0.018; 95% CI: 1.47–59.84), nematodes (OR = 15.5; *p* = 0.001; 95% CI: 3.11–76.70), cestodes (OR = 10.8; *p* = 0.025; 95% CI: 1.35–86.77), and being male (OR = 4.6; *p* = 0.036; 95% CI: 1.10–18.90) were associated with increased odds of LUAV RNA detection. The role of possible sexual and/or congenital transmission in the epidemiology of LUAV infections in rodents requires further study, along with the implications of possible helminth co-infection.

## 1. Introduction

Mammarenaviruses are a group of highly pathogenic and non-pathogenic, negative-sense, and single-stranded RNA viruses that infect mammals, including humans [1]. Their *genomes* are bi-segmented, comprising large (L) and small (S) segments, which are separated by an intergenic region [2]. Some mammarenaviruses cause severe haemorrhagic fevers [2]. During the early stages of disease progression, mammarenavirus infections may be confused for other febrile illnesses [3]. Treatment is usually symptomatic, but delayed diagnosis puts patients at risk of severe disease and death. Since the discovery of mammarenaviruses in Africa in 1969 [4], approximately 265 thousand people have died from mammarenavirus-related causes, mainly in West Africa where five thousand people die annually [3,5].

Mammarenaviruses belong to the family *Arenaviridae* and the genus *Mammarenavirus* [1]. They are grouped into two categories, New World (NW) and Old World (OW), based on phylogenetic, geographic, and epidemiological characteristics [2]. NW mammarenaviruses circulate in rodents belonging to the family *Muridae* and sub-family *Sigmodontinae* in South and North America except *Tacaribe* virus (TCRV), which is found in bats and ticks [1,2]. Viruses in this group include Chapare virus, Guanarito virus (GTOV), Junin virus (JUNV), Machupo virus (MACV), Sabia virus (SABV), and TCRV [1]. Meanwhile, majority members of the OW group are generally found in Africa [1]. Their reservoir hosts are rodents in the family *Muridae* and sub-family *Murinae* with the exception of Lujo virus (LUJV) whose host is unknown [1,6]. IPPY virus (IPPV), Lassa virus (LASV), LUJV, Mopeia virus (MOPV), Lunk virus (LNKV), Gairo virus (GAIV), Mobala virus (MOBV), Morogoro virus (MORV), and Luna virus (LUAV) are among the 22 mammarenavirus species found in Africa [1,7]. All 22 viruses are found south of the Sahara.

LASV and LUAV are the most widely distributed mammarenaviruses in Africa by geography. LUAV is found in south-eastern Africa [8,9,10,11,12], while LASV covers the majority of countries in West Africa [13]. LUAV and LASV, though distantly related, share the same reservoir host, the Natal multimammate mouse, *Mastomys natalensis* [14,15]. *M. natalensis* hosts seven mammarenaviruses (LASV, Gairo virus, Morogoro virus, Mobala virus, Mopeia virus, LUAV, and Dhati Welel virus) in Africa [7,9,15,16,17,18,19]. It has six divergent mitochondrial matrilineages forming two monophyletic clades, A and B. Clade A consists of subclades A-I, A-II, and A-III, while clade B has B-IV, B-V, and B-VI [13]. Each mammarenavirus appears to have a proclivity for specific subclades in Africa. For example, subclade A-I carries LASV [13] and B-IV is the reservoir host for GAIV and MORV [7,17], while B-VI carries MOPV and LUAV [11,19]. As viruses evolve and rodents migrate to new habitats due to human activities, infection across subclades may be inevitable [20].

The persistence of mammarenaviruses in a population of rodents from one generation to the next or across seasons appears to require a constant presence of a few chronically infected individuals [21]. What remains to be clarified particularly in wild rodents within their natural habitats is the organs that serve as sites of virus persistence. A previous field experiment involving *M. natalensis* naturally infected with MORV observed virus persistence in kidneys for 8 weeks [21]. More recently, persistence of LASV was observed in the lungs of *M. natalensis* for 12 weeks post experimental infection [22,23]. Testes were observed to keep LASV longer than ovaries, but statistically significant differences between mammarenavirus infections in male and female rodents have never been reported despite a number of attempts [7,18,19,21,22,23]. Aside from investigations in testicular tissues, none of the studies involving *M. natalensis* have reported on the dynamics of mammarenavirus in the seminal vesicles, either experimentally or in natural settings. Contrary to the situation in wild *M. natalensis*, prolonged secretion and persistence of LASV in semen were observed in humans in West Africa for close to nine months [24]. Another patient exhibited prolonged LASV-related cytotoxic T-cell responses in the absence of viraemia but high viral titres in semen [25].

The dynamics of transmission or persistence of any pathogen in the host or environment hinge on the complex interplay of factors related to the host, habitat (environment), and pathogen. Wet or high rainfall habitats appear to support the presence of some rodent-borne viruses such as LASV, Kodoko virus, Sangassou hantavirus, Sin Nombre hantavirus, and Puumala virus [26,27,28,29,30,31]. Whilst the reasons for this observation remain to be clarified, it has been suggested that soil type, moisture, organic matter, soil pH, and amount of salts in the soil may have direct or indirect effects on virus survival [32]. Theoretically, the phenomenon may also be a consequence of immunomodulatory activities of co-infecting helminths. There is strong evidence that some helminths alter the T helper 1 (Th1): T helper 2 (Th2) immune balance towards Th2 responses, which are humoral [33,34,35]. In turn, virus survival in the host may theoretically be prolonged in the absence of appropriate antiviral Th1 responses. Nonetheless, the relationship between mammarenaviruses and helminths remains hypothetical and needs further investigation.

In this study, we investigated the presence of mammarenaviruses in several tissue types and fluids (liver, kidney, lung, spleen, semen, and foetal tissues) obtained from small mammals in riverine and non-riverine habitats. Reverse transcriptase polymerase chain reaction (RT-PCR) was used for the initial screening of mammarenaviruses in all small mammals followed by in-depth investigations using metagenomic next-generation sequencing (mNGS) in *M. natalensis*, the only species in which arenaviruses were detected. The study sought to: (a) compare the prevalence between habitats; (b) compare the prevalence and virus-specific mNGS reads between tissue types; and (c) identify the risk factors associated with the presence of mammarenaviruses. Risk factors investigated included age, sex, habitat, season, and helminths (cestodes and nematodes).

## 2. Materials and Methods

### 2.1. Study Design, Study Site, and Rodent Trapping

**Figure 1:**
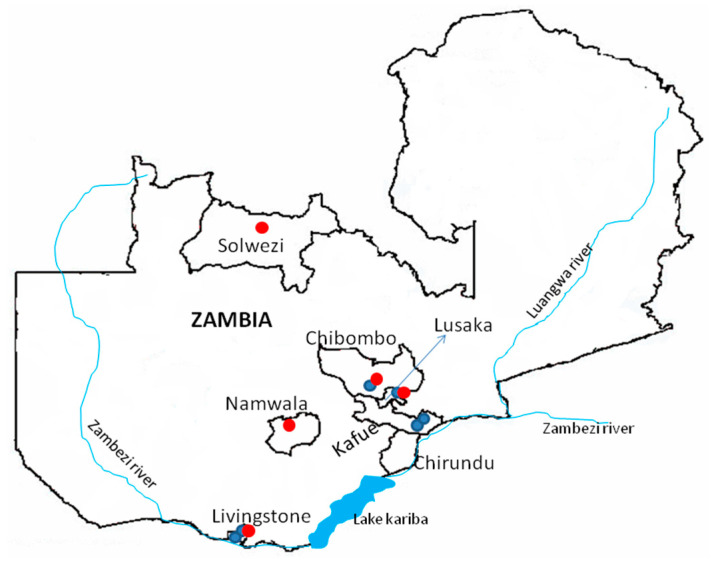
Map of Zambia (not drawn to scale) showing the rodent sampling sites (blue dots) in Chibombo, Lusaka, Kafue, and Livingstone districts. Areas where mammarenaviruses were previously found are indicated by red dots [9,14].

We conducted a cross-sectional study in selected sites in Zambia in Lusaka (15°26′16.511″ S, 28°26′22.174″ E), Kafue (15°54′38.271″ S, 28°52′32.64″ E), Livingstone (17°48.148″ S, 25°42.567″ E; 17°44.123″ S, 25°51.356” E), and Chibombo (14°58′6.12″ S, 28°26′9.93″ E) districts (Figure 1). The study areas included an inactive farm bordered by two streams in Lusaka, edges of a commercial maize farm and along a stream that bordered a soya bean farm in Kafue, a small-scale maize field and an undisturbed bush with a number of Mungongo (*Schinziophyton rautanenii*) trees in Livingstone, and a fallow farm in Chibombo. Study sites on the edges (0 to 100 m) of the streams were designated as riverine habitats, while those far away from any water bodies were defined as non-riverine habitats. All the non-riverine habitats had no nearby water bodies except one in Livingstone, which was over 1000 metres away from the Zambezi river. The rodents were captured between July 2019 and March 2022 using Sherman traps (H.B. Sherman, Inc., Tallahassee, FL, USA) set overnight and baited with peanut butter and pieces of cabbage or carrots.

### 2.2. Sample Collection

Captured small mammals were euthanised with diethyl ether. Their weights and tail length were measured. Age was determined by relative body size, sexual maturity (perforated vagina in the case of females and external visibility of testicles in males), and dried eye weight in milligrams (representative animals only) [7,19,36,37]. Animals were designated as juveniles (adolescents) or adults [38]. Thereafter, liver, kidney, spleen, and lung tissues were harvested. For male rodents, whole seminal vesicles containing semen were collected, and semen was carefully harvested into storage tubes. Foetuses were harvested from the uteruses of pregnant females. The samples were transported in portable cool boxes with ice packs from the study sites and kept in a −30 °C freezer until nucleic acid extraction.

### 2.3. Identification of Rodents

The identification of *M. natalensis* was conducted morphologically through ear, body, tail, and hindfoot length measurements and using the field guide to mammals of southern Africa and confirmed by cytochrome *b* metagenomic sequences. The rest of the small mammals were identified morphologically up to genus level using the field guide only. The study focused on identifying the rodents to species level, from which mammarenaviruses were detected.

### 2.4. Molecular Screening for Mammarenavirus RNA

A mixture of liver, kidney, spleen, and lung tissues per small mammal was homogenised in phosphate-buffered saline (PBS), while semen and foetal tissue from six foetuses were processed separately. Total RNA was extracted from the supernatant of the homogenised tissues using the QIAamp Viral RNA Mini Kit (Qiagen, Hilden, Germany) following the manufacturer’s instructions. Molecular screening for mammarenavirus RNA was conducted using the OneStep RT-PCR Kit (Qiagen, Hilden, Germany) under the following conditions: 30 min at 50 °C, 15 min at 95 °C, 45 cycles of 20 s at 95 °C, 30 s at 50 °C, 1 min at 72 °C, and a final extension step of 10 min at 72 °C as previously described [9,14]. Primer sequence pairs used in the study were reported previously [9]. The primers amplified a 1000 bp fragment, which was observed on 1.5% agarose gel stained with ethidium bromide. Positive PCR products were purified using the ZYMO DNA Clean-up and Concentration kit (Zymo Research, Irvine, CA, USA). Purified DNA was sequenced using a BigDye Terminator v3.2 Cycle Sequencing Kit on a 3500 Genetic Analyzer (Applied Biosystems, Foster City, CA, USA). Sequences were assembled and edited using GENETYX ATGC software version 7.5.1 (GENETYX Corporation, Tokyo, Japan).

### 2.5. Metagenomic Detection of Mammarenaviruses and Helminths

RNA samples from *M. natalensis* were subjected to metagenomic next-generation sequencing (mNGS) at the Chan-Zuckerberg (CZ) Biohub, San Francisco, United States of America (USA). We focused on *M. natalensis*, as it is currently the sole host of LUAV [11,19].

### 2.6. RNA Extraction, Library Preparation, and Next-Generation Sequencing

Total RNA extracted within three days before metagenomic sequencing was pooled with equal volumes of RNA later (ThermoFisher Scientific, Waltham, MA, USA) and transported to the CZ Biohub in San Francisco, United States of America. RNA was re-extracted using the QIAamp Viral RNA Mini Kit (Qiagen, Hilden, Germany). The protocol for library preparation was an adaptation of the NEBNext Ultra II RNA Library Preparation (non-directional) protocol (New England Biolabs, Ipswich, MA, USA). RNA was fragmented and spiked with External RNA Controls 103 Consortium collection (ERCC) (ThermoFisher, Waltham, MA, USA) as internal controls of library preparation errors, input for RNA mass calculation, reverse-transcribed into complementary DNA (cDNA), and ligated with adaptors, followed by digestion of bell-shaped adapters, barcoding, and final clean-up on a magnetic rack. The size and concentration of the library were determined using the 4150 Tapestation system (Agilent, MA, USA). Before loading onto the Illumina Novaseq 6000 (Illumina, 97 San Diego, CA, USA) for next-generation sequencing, the flow cell was washed to remove salts, followed by denaturing of the library with sodium hydroxide (NaOH) (0.2 N) and the addition of PhiX as a calibration control for the Illumina sequencing platform.

### 2.7. Determination of Luna Virus-Specific mNGS Reads per Million (rPM)

The number of mNGS reads aligning to any LUAV genome in the NCBI NT/NR database was determined for each positive sample from the Chan-Zuckerberg ID (CZ ID) metagenomics pipeline v7.0. The reads per million sequenced (rPM) metric is a scaled metric of abundance. “The rPM value provides a metric that enables comparison of relative abundances across samples sequenced to different total sequencing depths” [39]. The sum of reads was calculated according to tissue type based on the sex of the animals. The average coverage depth (percentage of reference genome in NCBI covered by reads) and the average depth of aligned contigs/reads over the length of the reference genome were calculated for each category of samples [39].

### 2.8. Quality Control Metrics and Detection of Pathogens

Metagenomic data generated from the Illumina Novaseq 6000 sequencer were analysed using the CZ ID sequencing pipeline, a cloud-based open-source bioinformatics platform. The pipeline identified viral and parasitic reads by interrogating the NCBI nucleotide and protein databases followed by a species-level annotation. Reads for each sample were subjected to quality control checks including a set of filters, which required nucleotide reads per million to be greater than 100 (NT_rPM > 100) and the nucleotide Z score to be one (NT_z score = 1). The Z-score statistic was computed due to the application of a background model to remove taxa that may have been prevalent in water controls and passed through filtration. The background correction model was obtained using negative water controls. Mammarenaviruses and helminths were identified from the fastq files using the CZ ID metagenomics pipeline designed to detect microbes from metagenomic data.

### 2.9. Phylogenetic Analysis of Detected Mammarenaviruses

The phylogenetic relationships of three LUAV detected in Zambia and reference genomes obtained from the GenBank were based on the full-length nucleotide sequences of the L segment of mammarenaviruses encoding the RNA-dependent RNA polymerase (RdRp). The full-length LUAV sequences from this study were obtained from mNGS data. The evolutionary history was inferred using the maximum likelihood method and the Tamura–Nei model in MEGA11 [40]. The tree with the highest log likelihood (−145,249.14) was shown. The analysis involved 26 nucleotide sequences. They included 3 sequences from this study, 21 OW mammarenavirus reference sequences, and 2 NW sequences (Machupo virus and Tacaribe virus) as outgroup sequences. There were a total of 6814 positions in the final dataset.

### 2.10. Statistical Analysis

Data analysis was conducted using SPSS Ver. 21 (IBM Corp, Armonk, NY, USA) and R Studio (RStudio core Team (2020)) statistical software to obtain descriptive results regarding the demographic characteristics of captured animals, the occurrence of mammarenaviruses, mNGS reads, and helminth co-infections. A bivariate approach through the Pearson chi-square test was used to determine whether the prevalence of mammarenaviruses in *M. natalensis* was associated with age (juvenile or adult), sex (male or female), season (dry or rainy), habitat (riverine or non-riverine), nematodes (present or absent), and cestodes (present or absent). The association was considered significant if *p* < 0.05. A stepwise binary logistic regression model was used to identify variables that may be predisposing factors for the occurrence of mammarenaviruses in *M. natalensis*. All variables with *p* < 0.25 in the bivariate analysis were included in the model. The generated model was tested for goodness of fit and predictability using the Hosmer–Lemeshow test and Omnibus test, respectively. Independent variables were considered to be risk factors when *p* < 0.05.

## 3. Results

### 3.1. Demographic Characteristics of Captured Animals

A total of 250 small mammals were captured in Lusaka, Kafue, Livingstone, and Chibombo districts of Zambia in riverine and non-riverine habitats (Table 1). A rodent, *M. natalensis* (confirmed identity via GenBank accession number OP778190), was the predominant species accounting for 72.8% (182/250) of all small mammals. *M. natalensis* has soft silky hair with pale grey underparts and grey-brown to almost black upper parts. Females have 12 pairs of nipples. The tail is finely scaled and almost equal to the length from the head to the end of the body. Other captured animals included *Gerbilliscus* sp., *Saccostomus* sp., *Rattus rattus*, *Crocidura* sp., *Arvicanthis* sp., *Mus* sp., and *Lemiscomys* sp.

### 3.2. Prevalence of Mammarenaviruses

LUAV was detected in 7.7% (14/182) of *M. natalensis* rodents (Table 2). All the positives were detected by both RT-PCR and mNGS except one, which was found by RT-PCR only. The virus was also detected in six foetuses obtained from a pregnant dam. The positive rodents were captured in Lusaka (12/14) and Kafue districts (2/14). Four out of the fourteen LUAV-positive animals were captured during the dry season in October, while ten were captured during the rainy season in November and December. The prevalence of LUAV was considerably higher in riverine (11.1%, 13/117) than in non-riverine (1.5%, 1/65) habitats.

### 3.3. Distribution of LUAV in Tissues

Three out of fourteen (21.4%, 3/14) positive rodents carried LUAV in semen only, while one (7.1%, 1/14) had the virus in both semen and mixed tissues (liver, spleen, kidney, and lungs). The rest (71.4%, 10/14) of the positive rodents had the virus in mixed tissues only. LUAV was also detected in six foetuses extracted from a single gravid uterus of one of the positive female rodents (Table 3). We used the reads per million sequences (rPM) metric [40], a scaled metric of abundance in the CZ ID bioinformatics pipeline v7.0, to compare the number of mNGS reads aligning to any LUAV sequence in the NCBI NT/NR database across the sample types. The number of LUAV-specific mNGS reads was 24- and 59-times higher in semen than in mixed tissues from male and female rodents, respectively. Mixed tissues from one foetus subjected to mNGS had 5285 more reads mapping to LUAV than all the tissues from female rodents.

### 3.4. Phylogenetic Analysis of LUAV Sequences

**Figure 2:**
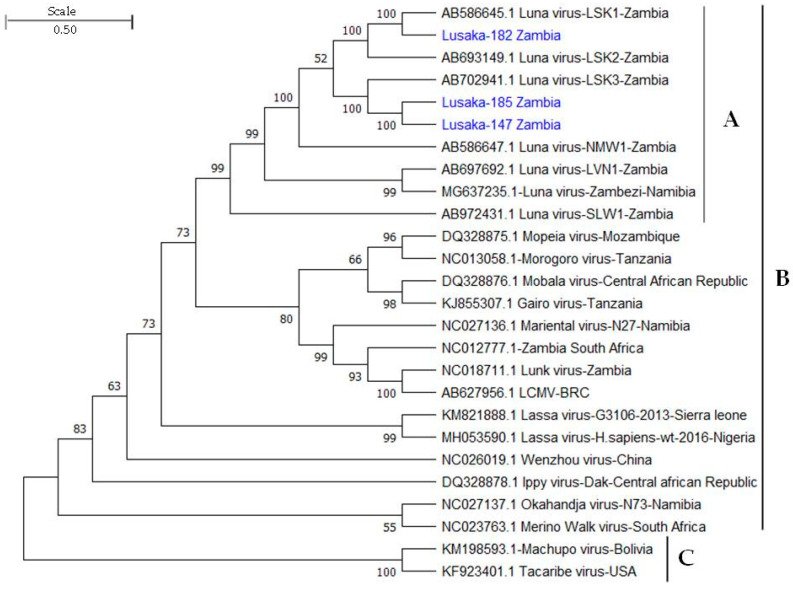
Phylogenetic relationships of Luna viruses detected in Zambia based on the nucleotide sequences of the L segment encoding the RNA-dependent RNA polymerase (RdRp). (A) Luna viruses, (B) OW mammarenaviruses, and (C) NW mammarenaviruses used as outgroup. The analysis involved 26 nucleotide sequences including three from this study (Lusaka-182, Lusaka-185, and Lusaka-147). In total, there were 6814 positions in the final dataset. Luna viruses detected in this study are in blue text, while the reference sequences are indicated by their GenBank accession numbers, virus names, and where applicable, country of origin. Bootstrap values ≥50% are shown at branch nodes. The scale indicates the number of substitutions per site. The analysis was conducted in MEGA11 [40].

Fifteen LUAV RNA sequences were detected (Table 4). Based on the full-length sequences of the well-conserved L gene [2] that encodes the RNA-dependent RNA polymerase (RdRp) protein, phylogenetic analysis was conducted. The resulting phylogenetic tree (Figure 2) included 3 LUAV sequences (i.e., Lusaka-185 Zambia, Lusaka-182 Zambia, and Lusaka-147 Zambia) from this study, for which we had obtained full-length sequences of the L gene, and GenBank accession numbers for the three viral sequences are OP778187, OP778188, and OP778189. The rest of the 12 LUAV sequences were not included in the phylogenetic analysis, as they were partial sequences and could not be translated into uninterrupted portions of the RNA-dependent RNA polymerase (RdRp) protein. The 12 sequences (Table 4) were either LUAV LSK-1 or LUAV LSK-3 strains, similar to the sequences for which we obtained GenBank accession numbers except one, which aligned to the LUAV NMW-1 strain. The sequences are available in Supplementary Material File S1.

### 3.5. LUAV–Helminth Co-Infections in M. natalensis

We detected helminths (cestodes and nematodes) in mixed tissues (liver, spleen, kidney, and lung) from 11.5% (21/182) of *M. natalensis* using mNGS (Table 5). One cestode species, *Hymenolepis microstoma* (OP779720), was detected in 3.8% (7/182) of the rodents. Six species of nematodes [*Caenorhabditis inopinata*, *Aonchotheca paranalis* (OP779770), *Calodium hepaticum* (OP779674), *Trichinella spiralis*, *Trichulis ovis* (OP800194), and *Pearsonema plica* (OP799550)] were detected in 7.7% (14/182) of the rodents. The sequences for *Caenorhabditis inopinata* and *Trichinella spiralis* were too short to obtain GenBank accession numbers (Supplementary Material File S1). Meanwhile, the majority (76.2%, 16/21) of *M. natalensis* infected with helminths came from riverine habitats compared to 23.8% (5/21) from non-riverine habitats. A total of 33.3% (7/21) of *M. natalensis* carrying helminth infections were co-infected with LUAV. Half (50%, 7/14) of *M. natalensis* that had LUAV were co-infected with helminths. The prevalence of LUAV was higher among rodents carrying more than one helminth species than those with single infections (Table 5).

### 3.6. Risk Factors for the Occurrence of LUAV in M. natalensis

Pearson chi-square (*χ*^2^) test was used to determine the relationship between sex, nematodes, cestodes, age, habitat, season, and infection with LUAV in *M. natalensis* (Table 6).

In a stepwise multivariate model, an insignificant Hosmer–Lemeshow goodness-of-fit statistic (*p* = 0.785) and Omnibus Test of Model Coefficients (*p* < 0.000) showed that our model fit the data and was better at predicting variance than the baseline model. The model explained 37.4% (Nagelkerke *R^2^*) of the variance in LUAV infections and correctly classified 92.3% of LUAV cases. Age, sex, nematodes, and cestodes were identified as risk factors for the occurrence of LUAV in *M. natalensis* (Table 7). Juvenile rodents were 9.4 (95% CI: 1.47–59.85) times more likely to have LUAV infection than adults. The odds of a LUAV infection were 15.45 (95% CI: 3.1–76.70) times greater for rodents infected with nematodes as opposed to those without nematode infections. Similarly, rodents infected with cestodes were 10.8 (95% CI: 1.36–86.77) times more likely to have LUAV than those without the infection. Male rodents were 4.6 (95% CI: 1.100–18.90) times more likely to carry LUAV than females.

## 4. Discussion

Using mNGS and RT-PCR, we detected LUAV mammarenaviruses in 14 *M. natalensis* rodents captured from the eastern outskirts of the city of Lusaka and for the first time in Kafue district. Ten years after it was first detected, LUAV is still circulating in the eastern outskirts of the city of Lusaka, while its presence in Kafue district may indicate a wider geographical distribution in Zambia than is currently known [9,14]. Hence, there is a need for expanded surveillance and characterisation of the zoonotic potential of these viruses, especially since LUAV circulates in *M. natalensis* in at least six countries in southern and eastern Africa [8,10,11,12,14].

In this study, the prevalence of LUAV was higher among juvenile rodents than adults. Our results are consistent with previous findings involving Gairo virus and Morogoro virus in *M. natalensis* in which infection was inversely related to age [7,17]. In another study, viral RNA was commonly detectable in juveniles, while mammarenavirus-specific antibodies increased with rodent age [21]. In natural settings, it is not completely clear how juvenile rodents acquire mammarenavirus infections. Congenital and horizontal transmission have been implicated [7,17,21]. We detected LUAV RNA in six fully grown foetuses obtained from a pregnant wild *M. natalensis*. The finding leaves open the possibility of LUAV’s capacity to cross the placenta to infect in utero foetuses. Considering that LUAV was detected in all the foetuses and the dam, this supports the idea that mammarenaviruses may have a high affinity for placental and uterine tissues [41]. Overall, the findings suggest that congenital transmission may contribute to the high prevalence of mammarenavirus infections in juvenile rodents frequently observed after the breeding season.

This is the first time that sex (in this case, male sex) has been identified as a risk factor for infection with any mammarenavirus among rodents despite a number of attempts in the past [7,17,18]. Male rodents are often at risk of infection due to risky behaviours such as fighting for mating partners and frequent movements during the breeding season [42]. However, the identification of male sex as a risk factor could also be attributed to our surveillance for mammarenaviruses in semen obtained from seminal vesicles. The approach enabled us to identify more positive male rodents, which otherwise could have been missed had we restricted our screening to the liver, lung, spleen, and kidney tissues, as is often done [7,8,9,10,11,14,15,16,17,18,27,43].

As far as we know, this is the first time that an OW mammarenavirus has been detected in semen and intra-uterine foetuses in wild-caught *M. natalensis* within their natural habitats. Previous observations involved testes, ovaries, and other tissues but were all laboratory-based [22,23]. The presence of LUAV in semen has important implications on virus persistence within the rodent host and transmission dynamics, which probably include the sexual route. Therefore, it is our considered view that seminal vesicles are potentially key sites for persistence of mammarenaviruses in *M. natalensis*. The findings also underscore the importance of screening several bodily organs and bodily fluids for mammarenaviruses in order to understand their distribution by tissue type as well as to increase the chance of finding positives. This view is consistent with the recent detection of LASV in the semen of a human patient who had persistent CD8 T-cell responses long after viraemia resolution [25]. The infectious potential of LUAV detected in semen remains unknown at this stage. However, LASV RNA obtained from human semen was infectious in experimental investigations in mice in a previous study [24]. Thus, our findings may benefit from future studies such as plaque assays focused on determining the infectious capabilities of LUAV RNA detected in semen and other tissues.

We observed a higher prevalence of LUAV in riverine habitats (11.1%, 13/117) compared to (1.5%, 1/65) in non-riverine habitats. This observation adds to emerging evidence which points to the subtle role that humid or riverine ecosystems may play in the prevalence of certain viruses [26,27,28,29,30,31]. For example, LASV infections in rodents in West Africa are 2.5 times higher in the rainy season than in the dry season [15]. Meanwhile, Kodoko virus and Sangassou virus (a hantavirus) in Guinea have so far been found in rodents living in wet habitats along river edges and swamps only [27,28]. Similarly, other viruses such as Blue river virus, Black Creek canal virus, El moro Canyon virus, and Limestone Canyon viruses in the United States of America (USA) were detected in rodents living in damp or wet habitats only as summarised previously [26]. It appears that wet or damp habitats may support virus maintenance in permissible hosts. However, this parameter turned to be marginally non-significant in this study when it was analysed in a multivariate model. Further investigations involving large sample sizes may be required.

Intriguingly, infection with either nematodes or cestodes was identified as a risk factor for LUAV infection in *M. natalensis*. This study did not determine active helminth infection through the detection of eggs in faeces or larva in tissues. However, it may be important for future studies to compare the dynamics of LUAV and associated immune responses in its host based on active or inactive helminth infections. None of the co-infected rodents showed macroscopic pathological disease. This was expected, as most OW mammarenaviruses except LCMV do not cause pathological disease in reservoir hosts [21]. Similar to the pattern of LUAV infections observed in this study, rodents from riverine habitats had a higher prevalence of helminths than in non-riverine habitats. We speculate that riverine habitats favour the transmission of both LUAV and helminths by supporting the survival of viruses and helminths (eggs) in such humid conditions [44]. Additionally, the effect of helminths on the likelihood of acquiring or having an infection with LUAV may be immunological in nature. Evidence from several studies shows that *Trichnella* sp. [33], *Trichulis* sp. [45], *Calodium hepaticum* [46], and *Hymenolepis* sp. [47] mute the Th1-induced inflammation driven by interleukin 2 (IL-2) and interferon gamma (IFNγ) in favour of Th2 responses characterised by IL-4, IL-5, and IL-10 [34]. Consequently, the host’s immune system becomes favourable for viruses to thrive persistently without effective immune defences. We also observed a high prevalence of LUAV among rodents that had multiple helminth infections compared to those with single infections (Table 5). There is a chance that a high burden of helminth infections, particularly simultaneous infection with multiple species of helminths, may have a greater impact on LUAV prevalence and dynamics in the host. This requires further investigation. Therefore, laboratory-based experimental infections are required to fully understand the immunological or non-immunological basis of LUAV–nematode/cestode interactions and their impact on virus clearance or persistence.

Besides the immunological assumptions about helminth–LUAV interactions, it is remarkable that *Hymenolepis microstoma*, *Caenorhabditis inopinata*, *Aonchotheca paranalis*, *Calodium hepaticum*, *Trichnella spiralis*, *Trichulis ovis*, and *Pearsonema plica* were detected for the first time in rodents in Zambia. We did not find evidence of these helminths in other mammals across Africa in the literature except Calodium hepaticum, which is widespread in rodents in Africa, Trichulis ovis in sheep in South Africa, and Hymenolepis microstoma in rodents in South Africa and Nigeria [48,49,50]. Calodium hepaticum, Trichnella spiralis, and Trichulis ovis are established zoonoses, while Hymenolepis microstoma was detected for the first time in humans recently [51]. The zoonotic potential of the rest of the helminths is not known.

## 5. Conclusions

The detection of LUAV mammarenaviruses in *M. natalensis* captured from riverine and non-riverine habitats in Lusaka district and for the first time in Kafue district in Zambia suggests that these viruses are still circulating in the country and that they may have an expanded geographic range. Age (juvenile), sex (male), and helminths (nematodes and cestodes) were identified as risk factors for LUAV infection in *M. natalensis*. Taken together, our study suggests that riverine habitats, helminths, and the presence of male rodents carrying LUAV in semen may support virus persistence/maintenance in rodent populations. Additionally, the transmission dynamics of LUAV among rodents probably involve congenital and sexual transmission.

## Figures and Tables

**Table 1:** Demographic characteristics of captured small mammals.

	Species	Location						Sex		Age	
		Riverine Habitat		Non-Riverine Habitat							
		LSK	KF	LSK	KF	LV	CH	M	F	Adult	Juvenile
Rodents	*M. natalensis* (n = 182)	101	16	0	5	27	33	82	100	169	13
	*Gerbilliscus* sp. (n = 27)	9	0	0	16	0	2	11	16	25	2
	*Saccostomus* sp. (n = 18)	0	0	0	5	0	13	6	12	13	5
	*Rattus rattus* (n = 13)	0	0	13		0	0	4	9	13	0
	*Mus* sp. (n = 1)	1	0	0		0	0	1	0	1	0
	*Arvicanthis* sp. (n = 1)	1	0	0		0	0	0	1	1	0
	*Lemiscomys* sp. (n = 1)	0	0	0	1			0	1	1	0
Shrew	*Crocidura* sp. (n = 7)	0	5	0	0	0	2	0	7	6	1
	Total (%)	133 (53.2)		117(46.8)				104 (41.6)	146 (58.4)	229 (91.6)	21 (8.4)

Abbreviations: LSK = Lusaka; KF = Kafue; LV = Livingstone; CH = Chibombo; M = Male; F = Female.

**Table 2:** Prevalence and distribution of LUAV in *M. natalensis* by habitat, sex, and age.

	Category	Characteristic	Prevalence
*M. natalensis* (n = 182)	Habitat	Riverine (n = 117)	11.1% (13/117)
		Non-riverine (n = 65)	1.5% (1/65)
	Sex	Male (n = 82)	12.2% (10/82)
		Female (n = 100)	4% (4/100)
	Age	Juvenile (n = 13)	23.1% (3/13)
		Adult (n = 169)	6.5% (11/169)

**Table 3:** Comparative mNGS reads for LUAV in semen, mixed tissues, and foetal tissues.

Sex	Sample Type	Number of Samples	Number of Reads Aligning to LUAV Genomes in the NCBI NR/NT Database, per Million Reads	Average Coverage Breadth **/Average Coverage Depth ***
Male	Semen	4	658, 925	77.8%/269.3x
	Mixed tissues *	6	27, 130	36%/5.1x
	Foetal tissues	1	16, 399	98.9%/50.1x
Female	Mixed tissues *	4	11, 114	55.9%/2.31x

* liver, spleen, kidney, and lung; ** Percentage of reference genome in NCBI covered by reads; *** Average depth of aligned contigs/reads over the length of the reference genome.

**Table 4:** LUAV isolates detected in *M. natalensis* in Zambia.

Luna Viruses	Reference (Accession Number)	Percent Identity
Lusaka-186 Zambia	LSK-1 (AB586645.1)	91.3
Lusaka-185 Zambia	LSK-3 (AB702941.1)	99.0
Lusaka-182 Zambia	LSK-1 (AB586645.1)	94.4
Lusaka-181 Zambia	LSK-3 (AB702941.1)	99.1
Lusaka-174 Zambia	LSK-3 (AB702941.1)	99.3
Lusaka-171 Zambia	LSK-1 (AB586645.1)	94.6
Lusaka-170 Zambia	LSK-3 (AB702941.1)	99.1
Kafue-167 Zambia	LSK-1 (AB586645.1)	91.8
Kafue-164 Zambia	NMW-1 (AB586647.1)	90.1
Lusaka-161 Zambia	LSK-3 (AB702941.1)	99.7
Lusaka-160 Zambia	LSK-3 (AB702941.1)	98.6
Lusaka-155 Zambia	LSK-3 (AB702941.1)	98.8
Lusaka-154 Zambia	LSK-3 (AB702941.1)	92.3
Lusaka-152 Zambia	LSK-3 (AB702941.1)	99.2
Lusaka-147 Zambia	LSK-3 (AB702941.1)	99.1

**Table 5:** LUAV–helminth co-infections in *M. natalensis*.

Helminth Infection Characteristic	Prevalence of Helminths (21/182)	Prevalence of LUAV (7/182)
≤1 helminth species (n = 17)	9.3% (17/182)	17.4% (3/17)
>1 helminth species (n = 4)	2.2% (4/182)	100% (4/4)
Cestodes (n = 7)	3.8% (7/182)	28.6% (2/7)
Nematodes (n = 14)	7.7% (14/182)	20.5% (5/14)

**Table 6:** Bivariate analysis of the association between LUAV and biotic/abiotic factors.

Variables	Characteristic	Luna Virus		*p*-Value
		Negative	Positive	
Sex	Male	71	10	0.035 *
	Female	97	4	
Nematodes	Negative	159	9	<0.0001 *
	Positive	9	5	
Cestodes	Negative	163	12	0.035 *
	Positive	5	2	
Age	Juvenile	10	3	0.031 *
	Adults	158	11	
Habitat	Non-riverine	64	1	0.020 *
	Riverine	104	13	
Season	Dry	88	4	0.087 *
	Wet	80	10	

* = Chi-square (*χ*^2^); *p* < 0.05 = Significant.

**Table 7:** Likelihood estimates for occurrence of LUAV in rodents by risk factor.

Risk Factor	Level	OR	95% CI	*p*-Value *
Age	Juvenile	9.38	1.47–59.85	0.018
	Adult	Ref		
Sex	Male	4.6	1.100–18.90	0.036
	Female	Ref		
Nematodes	Positive	15.45	3.11–76.70	0.001
	Negative	Ref		
Cestodes	Positive	10.85	1.36–86.77	0.025
	Negative	Ref		
Season	Rainy	2.07	0.509–8.44	0.309
	Dry	Ref		
Habitat	Riverine	7.94	0.85–3.56	0.068
	Non-riverine	Ref		

* Stepwise binary logistic regression; Significance level = *p* < 0.05; OR = Odds ratio; CI = Confidence interval; Ref = Reference category.

## Data Availability

Not applicable.

## Supplementary Materials

The following supporting information can be downloaded at: https://www.mdpi.com/article/10.3390/pathogens11111345/s1, Supplementary Material File S1: Luna mammarenavirus Zambia partial sequences. Supplementary Material File S2: Caenorhabditis inopinata and Trichnella partial sequences.
