# Supplementary material for: Luna Virus and Helminths in Wild Mastomys natalensis in Two Contrasting Habitats in Zambia: Risk Factors and Evidence of Virus Dissemination in Semen
Source: Pathogens. 2022 Nov 14;11(11):1345. doi: 10.3390/pathogens11111345 (PMC9697851; doi:10.3390/pathogens11111345)
Supplement: Supplementary file 1 [file pathogens-11-01345-s001.zip › pathogens-1995538-supplementary.pdf]

Supplementary Material File S1: Luna mammarenavirus Zambia partial sequences
>Luna mammarenavirus (NMW-1) Kafue-164-31 Zambia
GCGGTTGGTGTGGTGTTGATCTGCAGTTTTGTTGGCAGAGGGTACTTGCAAATGGGACAT
CTGTCGGATATTGTGAGAAGGAGTGTCAGACAGTTCATACATAAGAAATGGTCATAACAT
CTGACAAGGCCCCTTCTCTCAAACCAACAGCTCTTGCAGAACTCAGGTCCATGACCCGAA
GCATCAGGAATTAGCTCTGTCCTGGGTCCAGGGTCCTGGGCCACATGGGTTTGTTTTGAT
TGGGTTTTGCCCATTGTGAATGGTGGGGATTGTCCTGTGGTTGGGTGCCGGCCGGAGTCT
CCCTGGAGTGTGGGCTGCAAAATGAGTGCCTAGTAGATTAGCTTAAACTCAAATGATTTT
CAGATTTGCC

>Luna mammarenavirus (LSK-1) Lusaka-171-4 Zambia
GATCTTGGATGAGTTCAGGGACTGTACGGCGGGGGATTGCTGGCCGACTCTTTGGGGGCG
GTTGGTGTTTTGTTGATCTGCAGTTTTGTTGGCAGAGGGTACTTGCAGATGGGGCACCTG
TCGGAAATTGTGAGAAGCAATGTCAGACAATTCATACATAAATAATGGTCATAACATTTG
ACAAGACCTCTTCTCTCAAACCAGCAGCTTTTACAGAACTCAGGCCCATAGCCTGAGGCG
TCAGGTATCAGCTCCGTCCTGGGTTCAGAGACTTGGGCTTTGTGACCTTGCTTGGACTGG
GCTTTCCCCATTGTGGATGGTGGGGGAGTTCTGCGGACGGGCCCTACTTGAGGCTCCCT
CAGGGTGTGGGTGGCGAAACAGGCACCTAACTGGTTAGATTTCACTCAAATGAGCATCAA
AGTTGCC

>Luna mammarenavirus (LSK-3) Lusaka-160-5 Zambia
GCACAACACAGAGGAGTTGAGTGTTGAGGTGATCTTGCTCAATAAAGGTATCTTGAGTCC
TGGCCTGCCCTTTGTCGTGCCAGACGGTTTTAAACTTACTGGAAATGTATTGGTCTTGCT
AGAGTGCTTTGTTAGAAGCTCACCTTCAAACTTTGAGCAAAAGTATAATGAAGACTCTGT
AAAGTTGAGCTCCCTGAAACAAGACCTGCAGAGTGTGGGAATAACTTTGCTCCCTATAAT
TGATGGGAGGACAGTCTTTGATCATACTTTTATGCCGGAGTGGGCCAATGAAAGATTCAG
GTCGATTCTTTTCTCACTTCTCCAATTCGCTCAGGAATCATCGAGGATGTTGGAGGAATC
AGAGTACAGTAGACTCTGTGAATCATTGAGGAATGTAGAGGGCAAAAGATCAGGAATAGA
GAGTCTCAACATCCTTCAAGATTGTAGGGCAAGACATCACGAGGAAATTTTGAAATTGTG
TCACATGGGGATTGACAACTCCATAAACTCTATGGATGTCAAGCGAGAAATCATCAATGA
GTTCCAAGCTTTTAGAAATGGTGTAAGCTCTGGGTCAATCCCCAGACAATTTAGAAAAGT
TGACAAATCTGAGCTGTTAGAGTCTTTCTGTAAGATGTATCCTTCAGATTATGGCATGGA
GGCAGAAAGTGTAGGGAAGTTGAAGGGAGAATTCTTCATGCTATCTCCCACGATAACATT
ACTTTATGCTGACATTGATAGCACTGAAGAGTTAGGATCCAAGCACAGCTGTGTCCCAGC
TTGGAGGTCATTGTTGAACAAAATTAAAAGTCTCCACATACTTAATACTAGGAGAAAGAC
CTTGCTATTGTTTGATTCAATAATATTGCTGTGCCATGCCACTGACAAGGAAGTCGTTGG
CCACTTTGAAGAATCTGAGTGGCTCGGTTCTTCATTTCTGAGTGTGAACGATAGGCTCGT
CTCGTTGACTGCTACAAAAAGAGAATTGTATCAATGGATGGAAAGGAGAAGAATGAATCA
AGCAAAAAGGCCAAGCTCAAGATATGAGGATCGAGAACTGGTGTATTATAACCTTGTTAG
CTCCACTCTGAAGAAAGCTGAACAGGCATTAGCAGTTGCTAATTTGAAGTTTTC

>Luna mammarenavirus (LSK-3) Lusaka-161-7 Zambia
GTCCTGAGTAAAAATATTTACCTTTCAACTCATCATTCAAACAAACTTTCTCACAAATAA
CTTTGTAGTCATCATCAGATAAAAGTGACTGGTCAAAATCCTCTATCAAATGATTGGATA
ATTCTGATAAAATTAACTTCAAGTGCATTGGGTCATCAACAACCTTATGCAGATCATTTA
TGTCACCAGCATTGACTTTATCAAGTTTTTCATCACTTAGATTCCTGTAGCTCTCCATTA
CAGTCCTTACTGAATTTTTGATGTCTTCAAGATGAGAGGAGGCAGCACCACTAATCAACA
CTTCATCCAACTCTTCAGTAGACATTCCTTCACCAGAAAGGTTCTTATTGCCGATCACTA

GACTTGCAATCACTTTCATAATCTTGTAATCATATTCCTCTTTACTTAAAAAATATTTAC
TTTTTCTCTCAAATGTTTCTGACAATTGGCACACTGCAGCGGCAACAAGTTTGTCATAAT
CATAGTTCAACACCCTTTCACCATCAACATACTTATTTATGACCACACTTTTATTACTGG
CTAGATCCATCGCAGTTGCACAACCACTAAGGACCAAGGGGTCTTTAATTTCCCCTTTGA
ACTCTTGCTTGCTAAAAAGAGCACCATTATTAAATGATGATGTGACCAAGGATAAAATCT
TCTTTGAGA

>Luna mammarenavirus (LSK-3) Lusaka-155-54mo Zambia
ATTTGATAACAGTCGTCCTCAGTCTCTAGTGTTGGAGTCAGCTTATAAGGGAAGTCACTA
AGGTCATTCTTTTCATAATTGATAGATGGCATGATGCCTTCCACCTCAACTCCCATTATC
TTTTTAAAACTTAAAGTTGAGAATATGTTTTCCGACACACCATAATCAGAAAACTTCAAA
TTAGCAACTGCTAATGCCTGTTCAGCTTTCTTCAGGGTGGAGCTAACAAGGTTATAATAC
ACCAGTTCTCGATCCTCATATCTTGAGCTTGGCTTTTTTGCTTGATTCATTCTTCTCCTT
TCCATCCATTGATACAATTCTCTTTTTGTAGCAGTCAACGAGACGAGCCTATCGTTCACA
CTCAGAAATGAAGAACCGAGCCACTCAGATTCTTCAAAGTGGCCAACGACTTCCTTGTCA
GTGGCATGGCACAGCAATATTATTGAATCAAACAATAGCAAGGTCTTTCTCCTAGTATTA
AGTATGTGGAGACTTTTAATTTTGTTCAACAATGACCTCCAAGCTGGGACACAGCTGTGC
TTGGATCCTAACTCTTCAGTGCTATCAATGTCAGCATAAAGTAATGTTATCGTGGGAGAT
AGCATGAAGAATTCTCCCTTCAACTTCCCTACACTTTCTGCCTCCATGCCATAATCTGAA
GGATACATCTTACAGAAAGACTCTAACAGCTCAGATTTGTCAACTTTTCTAAATTGTCTA
GGGATTGACCCAGAGCTCACACCATTTCTAAAAGCTTGGAACTCATTGATGATTTCTCGC
TTGACATCCATAGAGTTCATAGAGTTGTCAATCCCCATGTGACACAATTTCAAAATTTCC
TCGTGATGTCTTGCCCTACAATCTTGAAGGATGTTGAGACTCTCTATTCCTGATCTTTTG
CCCTTTACATTCCTCAATGATTCACAGAGTCTACTGTACTCTGATTCCTCCAACATCCTC
GATGATTCCTGAGCGAATTGGAGAAGTGAGAAAAGAATCGACCTGAATCTTTCATTGGCC
CACTCCGGCATAAAAGTATGATCAAAGACTGTCCTCCCATCAATTATAGGGAGCAAAGTT
ATTCCCACACTCTGCAGGTCTTGTTTCAAGGAGCTCAACTTTACAGAGTCTTCATTATAC
TTTTGCTCAAAGTTTGAAGGTGAGCTTCTAACAAAGCACTCTAGCAAGACCAATACATTT
CCAGTAAGTTTAAAACCGTCTGGCACGACAAAGGGCAGGCCAGGACTCAAGATACCTTTA
TTGAGCAAGATCACCTCAACACTCAACTCCTCTGTGTTGTGCTCACAACCATTAGCCTCA
CAACTGTCCAGTTCTATACACAAAGAGAGCAGCTTGAGGCCCTCAATCAAGAGGTTGTTT
GGCTCAGACTGGATCAGGAAGTTTAATTTTTGCCTGGATAATCTCTGATCGTTGGCTAAG
TATTTAGAAATTAGATCCTTCGCCTCACACACACTTGTCTCCATTATAAG

>Luna mammarenavirus (LSK-3) Lusaka-152-6mo Zambia
GCAATGTGTAATCCTTGTGATTACTTTCCCTTGAAACCCACAATCACAAAGTTACTAGAG
GATAAAGTGGGGTTCAACCACATTATACACTCATTCAGAAGACTTTACCCGTCACTATTT
GAAAGGCACTTGTTGCCTTTTATGAGTGATTTGGCTTCCACAAAGATGAAGTGGTCCCCA
AGAGTGAAATTTCTTGACCTGTGTGTTGTCTTGGACGTCAATTGTGAAGCAATGTCCCTC
ATTAGCCATGTGGTCAAGTGGAAGCGAGAGGAGCATTACATTGTTCTATCATCAGAGTTA
TCAGAAGCTCATGATCGATCTCATGTATCTTTGGTGGAAGAAAGGGTGGTTAGTACCGAA
GATGTGGCAAAGAATTTCTTAAGGCAAGTCTTTTTTGAGTCCTTCATTAGACCCTTTGTT
GCGACAAGCAGAACATTAAGATCTTTTACCTGGTTTCCTCACAGGACGTCACTCCCTGCT
TCTGAGGGACTAAGCCTTCTCGGCCCATTT

>Luna mammarenavirus (LSK-3)Lusaka-181-2 Zambia
AGGGTTTTGTTCAAATGTCAAAGTGCAGGTCATCAAAGATGCACAAGCACTTTTACATGG
CTTAGACTTTTCAGAGGTTAGTAATGTCCAAAGGCTGATGAGAAAGGAAAAAAGAGATGA

CTCAGATCTCAAGAGACTGAGAGACTTAAATCAAGCAGTCAACAATCTTGTTGAACTTAA
ATCTTCACAACAGAAGAATGTGTTACGTGTGGGGACATTATCATCAGATGACCTTCTGAT
ACTGGCCGCAGATCTTGAGAAACTCAAATCAAAGATCACTAGATCAGAAAGACCTTTAAC
AGCTGGTGTTTACATGGGGAATCTGACAGCTCAACAGCTGGACCAAAGGAAAGCTCTCCT
CCAGATGATTGGCATGGGGCAGAGAAACTTAAATCCAATGGTGAGAGGTGACGGTATAGT
GAGAGTGTGGGATGTAAGAAACCCGGAACTACTCAACAATCAGTTTGGAACCATGCCAAG
CCTAACTGTTGCATGCATGTGCAAACAAGGTCAAGTTGACCTGAACGATGTCATTCAATC
TCTTAGTGATTTGGGGCTGGTGTATACAGCAAAATATCCAAATACATCAGATCTTGACAA
GTTAGCTCAAACTCATCCCATTCTCAATTTAATAGATGTGAACAAGAGTGCCATTAATAT
CTCTGGGTACAACTTCAGCCTGAGTGCAGCTGTTAAAGCAGGTGCAAGTTTGCTGGATGG
GGGGAACATGCTTGAGACTATCAGAG

> Luna mammarenavirus (LSK-3) Lusaka-186-8 Zambia
CAGTGATCAATTTTGTAAGCCGGAGCCTGTGTGGATAGTTGATGCGGACAACCTATCAGA
ATTTCTTGAGGATCCCAAATCAATTGGGCAGGTCGTTAGGCTCAATGTTAAACTAAGTGA
GGGTGTCACAGACCTCACAGAATATGATTTTTTGCACATAGGTCCAGATCCTGAGCCTTT
GCCTCTGGTTGTGGAGGATGGATACTTGGTAGAAGGCAAGAAGAAAGTTT

> Luna mammarenavirus (LSK-1) Kafue-167-41 Zambia
CTTTAGATGAATTTTGGGAGGCTCTCCAATCATTGCCGGGCGGATCCACTCAGCTCACAT
TTTCGGTTTTCTTTACGATTAAGAGTAAAGGGGAGACCCTGAGTGAAGAATTTGCGTTTC
ATGTCGAAACTTGGGGCATGATAGACCACACAGGTTCCTTTGTATTAAGTAGGTTAGAAG
TGCTATACAGTGGCCAAGTGCACAGGTCTCTGTTATTGGATTGTTGGAGATTAG

> Luna mammarenavirus (LSK-3) Lusaka-170-3m Zambia
TGGCCGGTTTGTTGCAGAGTTCAAGTCAAGGTTTTATGTTTGGGGTGATGAAGTCCCTCT
ACTGACGAAGTTTGTCGCAGCAGCCTTACACAATGTTAAGTGTAAAGAACCACATCAGTT
GGCAGAGACCATTGACACAATCATTGACCAGTCTGTGGCAAATGGGGTACCTGTGAAACT
GTGTAACCTGATACAACAACGAACCTTGGATCTTCTTAGGTATGCACAATATCCGATTGA
CCCGTTTTTAATGTATTGTGAC

> Luna mammarenavirus (LSK-3) Lusaka-174-21m Zambia
CTCTCACATCAGCTCAATTTTAGACATGGGGCAAGGTATCCTTCATAATGCCTCTGACTT
TTACGGACTTATAACAGAAAAATTCATCAACTATTCCATTGGTTTGCTTTATGAAGGGAC
TCTAAAATCATACACTTCTAGTGATGACCAGATCTCTTTATTCGATCACAAGTTGACTAG
CCTGTTAGACAAAGATCCTAATGAATTTGAGTACATCTTAGAGTTTCACAATTATTTGAG
TGACAGATTGAATAAAT

> Luna mammarenavirus (LSK-3) Lusaka-154 Zambia
GCGCGCTTTACAAGTCACTCATTTAATAGGTGAGGTGCCAACTAAGATGGGGCAAATAAT
CACCTTCTTTCAAGAGGTGCCACACATAGTGGAGGAAGTGATGAACATAGTCCTCATCAC
TCTATCCCTTCTTGCGATTCTCAAAGGAATATACAACCTGGCGACATGTGGATTGCTGGG
CTTGATTTCATTCCTATTTCTGTGTGGGAGGTCCTGCACATTGGTATACAAGGACAACTA
TGCTTTGAATAGTGTGGAACTCGATATGTCTACCCTGAATTTCACAATGCCGCTGTCTTG
TTCCAAGAACAATACCCACCATTACATTCAGACCTACAACGATTCTGGTTTGGAGCTCAC
ACTGACAAACATGTCTATTTTAAATCACAAGTTCTGCAACCTCTCTGATGCACATAGAAG
AAACCTGTATGACCACACCCTAATGTCCGTGGTGACAACCTTTCACTTGAGCATCCCCAA
TTTCAACCAGTATGAATCCATGGCTTGTGACTTTAACAGCGGGAAGATAACTGTTCAATA

Supplementary Material File S2: Caenorhabditis inopinata and Trichnella partial sequences
>Caenorhabditis inopinata Lusaka-157 Zambia
CTTTGAGATTGATTGAACTGGTGAGTCAATTCAACTGTAGAATCACCTCGTTTCCAGCCG
ACAATGATGGCTTCTCGTAGGTCTACAGTGAGGGATTTCCT

>Caenorhabditis inopinata Lusaka-186 Zambia
TCTCCCGGAATTCGTTGGTTGGATATCAAATTGGAAGATGAATTCAATATCATATTTTTCTTACAG
AACAGTCGAAGATCTACTAGTTACCAACTATCACAAACAG

>Trichinella spiralis Lusaka-155/171 Zambia
AACAGCAGTTGGACATGGGTCAGTCGATCCTAAGAAAACGGCGAAAGCTTGTTCGAATTTGCGA
CATGAATTGTAAGACTGAATAATTATTTGTGTGTGTGTTGTGGTGGAGGCGATTGTGTCG
